# Do Maternal Self-Criticism and Symptoms of Postpartum Depression and Anxiety Mediate the Effect of History of Depression and Anxiety Symptoms on Mother-Infant Bonding? Parallel–Serial Mediation Models

**DOI:** 10.3389/fpsyg.2022.858356

**Published:** 2022-05-26

**Authors:** Ana Filipa Beato, Sara Albuquerque, Burcu Kömürcü Akik, Leonor Pereira da Costa, Ágata Salvador

**Affiliations:** ^1^Digital Human-Environment Interaction Lab (HEI-Lab), Lusófona University, Lisbon, Portugal; ^2^Research Center in Neuropsychology and Cognitive Behavioral Intervention, Faculty of Psychology, University of Coimbra, Coimbra, Portugal; ^3^Department of Psychology, Faculty of Languages and History-Geography, Ankara University, Ankara, Turkey

**Keywords:** bonding, depression, anxiety, self-criticism, mothers, postpartum

## Abstract

**Introduction:**

History of depression symptoms, including before and during pregnancy, has been identified as an important risk factor for postpartum depression (PPD) symptoms. This condition has also been associated with diverse implications, namely, on the quality of mother–infant bonding. Moreover, the role of self-criticism on PPD has been recently found in several studies. However, the link between these factors has not been explored yet. Furthermore, anxiety symptoms in postpartum has been less studied.

**Methods:**

This study analyzed whether the history of depression symptoms predicted mother–infant bonding, *via* self-criticism and PPD symptoms. The same model was repeated with a history of anxiety and postpartum anxiety symptoms. A total of 550 mothers of infants <24 months old participated in this cross-sectional study and answered an online survey.

**Results:**

Through a parallel–serial mediation model, the results show that in a first step, self-criticism dimensions of inadequate-self, hated-self, and reassuring-self, and in a second step, PPD symptoms, mediate the relationship between the history of depression symptoms and mother–infant bonding. However, the relationship between the history of anxiety symptoms and bonding is not mediated by all the considered chain of mediators, being only mediated by one of the self-criticism dimensions, inadequate self.

**Conclusions:**

The current study confirmed the association of history of both depression and anxiety with mother–infant bonding. While in the case of history of anxiety symptoms, the relation was only mediated by inadequate self-dimension of self-criticism, in the case of history of depression symptoms, the relation was mediated by self-criticism and postpartum depressive symptoms. The buffering effect of reassuring-self on bonding and negative affect was also evidenced. Psychological and preventive interventions should address this evidence to target interventions for mother–infant bonding problems in accordance with previous and actual current maternal risk factors.

## Introduction

With the pregnancy and the birth of a child, mothers face important physiological, psychological, and social challenges and, for some, these periods may entail substantial emotional pain and distress (Staneva et al., 2015). Depression affects 7–25% of women during the antenatal period (Gavin et al., 2005; Field et al., 2006; Dubber et al., 2015; Staneva et al., 2015), and 11–20% of women during the postpartum period, making postpartum depression (PPD) the most prevalent clinical condition after childbirth and an important public health problem (de Tychey et al., 2005). Also, it is estimated that 30–50% of cases will last 6 months or more and 25% of mothers will continue to be depressed 1 year later (National Institute for Health and Care Excellence, 2003). Recent studies had even highlighted the stable and chronic trajectory of these symptoms until at least 24 months after childbirth (Kiviruusu et al., 2020). The PPD is a relatively common postpartum complication with a global pooled prevalence of 17.7% with significant heterogeneity across different nations (Hahn-Holbrook et al., 2018). The PPD is characterized by disabling symptoms such as persistent sadness, low self-esteem, anxiety, irritability and sleep/appetite alterations, dysphoria, loneliness, emotional lability, insomnia, confusion, guilt, and suicidal ideation (Letourneau et al., 2011). The previous psychopathology, specifically the history of prior depression, has been highlighted as one of the most important PPD risk factors (Robertson et al., 2004). More importantly, PPD may not only impact a mother's self-care but also the ability to cope with the care of the child.

In addition to the postpartum depressive symptoms, the symptoms of anxiety symptomatology are also common in postpartum and during pregnancy. Although it remains less studied than PPD and is largely underdiagnosed (Sawers and Wong, 2018), the two conditions are mostly comorbid (Hofmeijer-Sevink et al., 2012; Kubota et al., 2014; Takehara et al., 2018). Anxiety symptoms overlap with PPD, but they are distinct diagnostic entities; therefore, screening for the postpartum mental difficulties should include not only depression but also anxiety (Nakić Radoš et al., 2018). In addition, despite the inconsistency of the studies concerning the prevalence of anxiety during the postpartum period, some studies suggest that 20–25% of women have anxiety disorders during pregnancy, and 15–20% in the postpartum period. When anxiety symptoms in general are explored (trait anxiety), these rates increase to 25–33% during pregnancy, 17–22% in the postpartum period, and 15–33% in the late postpartum period (e.g., Grant et al., 2008; Dennis et al., 2013), highlighting the long-term duration of anxious symptoms. Despite the high prevalence of anxiety problems in the postpartum period, there is a lack of studies of this condition (Matthey et al., 2003; Wenzel et al., 2003; Tietz et al., 2014). The previous psychopathology, particularly the history of anxiety, might be highlighted as one of the most important postpartum anxiety risk factors, characterized by autonomic arousal, skeletal muscle effects, situational anxiety, and subjective experience of anxious affect (Lovibond and Lovibond, 1995). Matthey et al. (2003) found that a previous history of anxiety disorder posed a major risk factor for anxiety at 6 weeks postpartum.

Bonding is a complex phenomenon that represents numerous stages in the development of the relationship between mother and baby (Hill and Flanagan, 2020). Maternal bonding, which is believed to develop during pregnancy or immediately after childbirth as a dynamic construct (Bicking Kinsey and Hupcey, 2013) and continues to improve in the first months of the infant's life (Muzik et al., 2013), is defined as “an affective state of the mother,” and corresponds to emotions and cognitions of a mother toward her baby (Billings, 1995; Klaus et al., 1995; Bicking Kinsey and Hupcey, 2013). A maternal bonding refers to the emotional messages and actions a mother displays to her baby, while attachment refers to the caregiver's closeness and commitment that enables a baby to form a positive connection with them (Goulet et al., 1997; Redshaw and Martin, 2013). Impaired mother–infant bonding includes delays in mothers' emotional responses toward their infant, anger, hostility, indifference, and rejection (Brockington et al., 2001, 2006). The mother–infant bond attracts a lot of attention, not only having an important role in the baby's wellbeing but also in the child's cognitive and emotional development (e.g., Tamis-LeMonda et al., 2001; Cirulli et al., 2003).

Considering the studies examining the relationship between mother–infant bonding problems and PPD and postpartum anxiety, it can be said that a poor parental mental health is one of the main risk factors for impaired parent–infant interactions that may lead to adverse effects on bonding (Reck et al., 2004; Parfitt and Ayers, 2009). Although disorders of mother–infant bonding are seen even in healthy postpartum mothers (Vengadavaradan et al., 2019), research on potential risk factors related to mother–infant bonding has focused on postpartum maternal mental health, in particular on PPD (Handelzalts et al., 2021). Research to date provides substantial evidence that both antenatal depressive symptoms (Kolk et al., 2021) and PPD measured early after childbirth could predict bonding difficulties until 1 year after childbirth (Brockington et al., 2006; Moehler et al., 2006; Muzik et al., 2013; Nonnenmacher et al., 2016; Tsuchida et al., 2019; Kasamatsu et al., 2020; Handelzalts et al., 2021). Depression in pregnancy and after birth could have an adverse impact on women, their children, and their relationships (World Health Organization, 2008). Other studies demonstrated that not only PPD but also depressive symptoms are related to impaired mother–infant bonding (Moehler et al., 2006; Edhborg et al., 2011; Hairston et al., 2011; Tietz et al., 2014; Dubber et al., 2015; Garcia-Esteve et al., 2016; Kasamatsu et al., 2020; Nakić Radoš et al., 2020). According to some studies addressing multiple risk factors, both the history of depression (Nonnenmacher et al., 2016; Badr et al., 2018) and depression in pregnancy (Ohoka et al., 2014; Daglar and Nur, 2018) along with PPD, have been associated with impaired mother–infant bonding. Similarly, in one study, clinically defined maternal depressive disorder during pregnancy is shown to negatively impact maternal–fetal bonding (McFarland et al., 2011), suggesting that the basis for poor mother–infant bonding in PPD may have roots in pregnancy (Lefkovics et al., 2014). On the other hand, another study showed that the maternal depression during pregnancy was not significantly associated with mother–infant bonding (Brassel et al., 2020).

In addition to the symptoms of depression, anxiety-related problems also have effects on bonding. Several research projects have investigated the link between postpartum anxiety and mother–infant bonding (e.g., Edhborg et al., 2011; Tietz et al., 2014). Tietz et al. (2014) found that mothers with postpartum anxiety disorder reported significantly lower bonding than healthy mothers. Further analysis showed that it was not a diagnosis of anxiety disorder itself but concurrent subclinical depressive symptoms together with avoidance of anxiety-related situations, that predicted lower mother–infant bonding. Similarly, in rural Bangladesh, maternal anxiety symptoms were positively associated with mother's emotional bonding (Edhborg et al., 2011). In another study, the higher levels of postpartum-specific anxiety were related to impaired overall bonding scores, subscales of impaired general bond, rejection and anger, and infant-focused anxiety across the first 6-months of life (Fallon et al., 2021). Feldman et al. (1997) stated that an increased anxiety during prenatal and postnatal periods seem to interfere with the mother's ability to bond and interact sensitively with the child. In addition, several studies indicate the significance of maternal anxiety on mother–infant bonding behaviors, the mother–infant relationship, and mother–infant interaction (e.g., Manassis et al., 1994; Nicol-Harper et al., 2007; Feldman et al., 2009; Kaitz et al., 2010).

Self-criticism was also considered as a mediator in this study. Despite the increasing attention in literature, self-criticism has been scarcely studied in the context of adaptation and transition to motherhood but represents a promising mechanism to comprehend postpartum distress. Self-criticism refers to a persistent and intense form of internal dialogue that involves self-scrutiny and expression of hostility and contempt toward the self (Whelton and Greenberg, 2005; Kannan and Levitt, 2013). There are two different forms of self-criticism, known as the “hated-self” and the “inadequate self.” The first one focuses on harsh self-loathing and the desire to remove unwanted aspects of the self with the function of self-persecution. The second one focuses on shortcomings or failures, with the function of self-correction (Gilbert et al., 2004). Referring to the relationship between self-criticism and history of depression and anxiety, both forms of self-criticism, but especially hated-self, have been consistently associated with psychopathology (Castilho et al., 2017; Kotera et al., 2021). For example, some studies showed that the high levels of self-criticism have been consistently shown to be a risk factor for the development of depression (e.g., Ehret et al., 2015; Zhang et al., 2019). Other research on female adolescents demonstrates that self-criticism successfully predicted the first onset of nearly all depressive and anxiety disorders (Kopala-Sibley et al., 2017). However, in their study on student samples, McIntyre et al. (2018) did not find that self-criticism predicted future levels of anxiety. On the other hand, self-reassurance (i.e., the ability to focus on one's positive aspects and be compassionate toward the self when things go wrong) functions as a buffer against self-criticism and therefore appears to be a protective factor against the development of psychopathology (Gilbert et al., 2004; Werner et al., 2019).

In the postpartum period, women seem to be particularly prone to self-criticism (Brassel et al., 2020), given the changes in maternal identity and the lack of control and autonomy accompanying motherhood (Priel and Besser, 1999; Brassel et al., 2020). Concerning the association between self-criticism and PPD and postpartum anxiety, such thinking style and emotions may heighten women's vulnerability to postpartum depression and anxiety symptoms. Although research on the effects of self-criticism on postpartum depression symptoms is still limited, existing studies have shown that postpartum depressed women presented higher levels of self-criticism compared to non-depressed women, and both depressed and non-depressed mothers' self-criticism was related to state anxiety (Vliegen and Luyten, 2009). In addition, self-criticism was strongly and positively associated with postpartum depressive symptoms (Vliegen et al., 2006; Besser et al., 2007). However, it is important to consider that self-criticism is described as a transdiagnostic factor, rather than a specific cognitive appraisal from depression, given that it seems prevalent in other psychological disorders (Luyten et al., 2007), such as anxiety disorders (Vliegen and Luyten, 2009; Castilho et al., 2014), stress (Luyten et al., 2011; Mandel et al., 2015), and social anxiety (Shahar et al., 2015; Lazarus and Shahar, 2018).

Self-criticism may also be associated with difficulties in mother–infant bonding. Beebe et al. (2007) found that at 4 months, self-critical mothers displayed less gaze and facial coordination with their infant and poorer infant attachment security at 20 months. Mothers may interpret infant signals and behavior as a reflection of their self-inadequacy and may therefore interact less with the child (Kaminer et al., 2007) or reduce their involvement in caregiving (Reizer and Mikulincer, 2007). In addition, self-critical mothers may project onto the infant feelings of resentment due to the loss of control and autonomy imposed by motherhood (Priel and Besser, 1999; Casalin et al., 2014; Brassel et al., 2020).

In summary, although there is evidence of the maternal history of depression and other forms of psychopathology as predictors of PPD and the quality of mother–infant bonding, studies have rarely included self-criticism as a mechanism explaining this link. Furthermore, although many studies have studied categorical diagnoses of PPD and anxiety disorders, they have not been able to capture the vast range of severity and intensity of depressive and anxious symptoms across the diverse stages of the postpartum period (Gorham, 2020). Furthermore, the history of maternal anxiety symptoms has been poorly studied in the literature on postpartum (in)adaptation. As such, this study aimed to analyze the association between maternal history of depression and anxiety symptoms, and mother–infant bonding, through self-criticism and levels of depressive and anxiety symptoms. According to previous studies, we first hypothesized that having a history of depression symptoms would predict less mother–infant bonding through higher levels of self-criticism and higher PPD. Although anxiety has been less explored in literature, given its high prevalence in the postpartum period and its relation to self-criticism, we also hypothesized that the history of anxiety symptoms affects bonding, *via* self-criticism and postpartum anxiety.

## Materials and Methods

### Participants

The sample included 550 Portuguese mothers, aged 18–48 years (*M* = 32.76, *SD* = 5.06). Most mothers had completed a university degree, were married/living with their partner, and were not currently on maternity leave. Concerning delivery mode, 63.7% reported giving birth vaginally and 36.3% giving birth through cesarean (programmed or emergency). The percentage of mothers who reported a chirurgical mode of delivery (planned or emergency cesarean section) is in line with the average of Portuguese national statistics for cesarean, that is, 36% (INE, 2021). Infants were aged between 2 weeks and 24 months (*M* = 8.57 months, *SD* = 6.51). The participants' demographic characteristics are shown in Table 1.

**Table 1:** Sociodemographic characteristics of the sample.

	***M* (*SD*)/*n* (%)**
Mothers' age (years)^a^	32.76 (5.06)
**Mothers' education**	
Basic/secondary education	206 (37.5%)
University degree	342 (62.1%)
Other	2 (0.4%)
**Mothers' marital status**	
Single	33 (6.0%)
Partnered without living together	12 (2.2%)
Married/partnered and living together	499 (90.7%)
Divorced/separated	6 (1.10%)
**Household monthly income**	
Less than €1583	283 (51.5%)
1€583 or above	267 (48.5%)
**Gestational complications**	
Yes (e.g., high-risk pregnancy, maternal health problems, fetus'/baby's health complications)	313 (56.9%)
No	237 (43.1%)
**Mode of delivery**	
Vaginal delivery	245 (44.5%)
Instrumental vaginal delivery	106 (19.2%)
Planned cesarean section	96 (17.5%)
Emergency cesarean section	103 (18.8%)
**Gestational age**	
Preterm childbirth (<37 weeks)	33 (6.0%)
Post-term childbirth (37 weeks or more)	517 (94.0%)
**Currently in maternity leave**	
Yes	237 (43.1%)
No	312 (56.9%)
Infants' age (months)^b^	8.57 (6.51)
**Number of children**	
1	349 (63.5%)
2	163 (29.6%)
3 or more	33 (6.4%)
Missings	3 (0.5%)

a *One participant did not report her age.*

b *Eight participants did not report the age of their infants*.

The inclusion criteria were as listed in the following: (a) To be a biological mother of one baby <24 months old, excluding twins; (b) to have conceived the baby in a context of a heterosexual relationship; (c) to have adequate knowledge of the Portuguese language to be able to complete questionnaires; and (d) to be 18 years and over. Mothers completed an online survey. Informed consent was obtained from all the women before they answered the protocol. Among the 556 mothers who participated, six questionnaires were excluded because one or more measures left more than 20% of the questions incomplete. In sum, 550 mothers were included in this study. Based on the cut-off points used by Lovibond and Lovibond (1995), mean scores indicated normal levels of depression and anxiety among participants.

### Measures

#### Sociodemographic and Clinical Background Questionnaire

Sociodemographic and clinical background questionnaire was applied to obtain information about the sociodemographic characteristics (e.g., age, relational status, academic degree, professional situation, cohabitation, number/ages of children, and type of delivery), pregnancy, breastfeeding, problems during and postpartum of the participants. This questionnaire also included several questions related to risk factors for PPD symptoms and (in)adaptation, such as maternal psychopathology; medical support during pregnancy, childbirth, and postpartum; childbirth experience; baby's temperament; distress during pregnancy; partner's distress during pregnancy/in the present; and body image. For this study, four items assessed the history of depression and anxiety symptoms, respectively (“Before pregnancy, did you feel sad or depressed often?” and “During pregnancy, did you feel sad or depressed often?” “Before pregnancy, did you feel anxious, nervous and/or tense often?” and “During pregnancy, did you feel anxious, nervous and/or tense often?”). The two items assessing the history of depression symptoms were aggregated and entered in the analyses as independent variables. The same procedure was repeated to obtain the score from history of anxiety symptoms. Each of these items were measured on a 5-point Likert scale, ranging from 1 (“I strongly disagree”) to 5 (“I strongly agree”). The use of this scale was to capture a dimensional continuum rather than a simplistic and dichotomic answer (yes or no). Good correlations were found between the items that measured the history of depression symptoms (*r* = 0.60, *p* < 0.001) and the history of anxiety symptoms (*r* = 0.57, *p* < 0.001).

### Forms of Self-Criticizing and Self-Reassuring Scale

The Portuguese version of the forms of self-criticizing and self-reassuring scale (FSCSRS) (Gilbert et al., 2004; Castilho et al., 2015) consisted of 21 items to assess how critical/attacking or how supportive and reassuring participants are when things go wrong. The scale has three subscales. The subscale of “Inadequate-self” (10 items) assesses the feeling of inadequacy of the self in the face of failures, obstacles, and mistakes (“I think I deserve my self-criticism”). The subscale of “Hated-self” (three items) evaluates a more destructive response, based on self-loathing, anger, and aversion to failure situations, characterized by a disliked relationship with the self and by a desire to hurt, chase, and assault the self (“I get so angry with myself that I want to hurt myself or harm myself”). The subscale of “Reassuring-self” (8 items) assesses a positive, warm, comforting, and compassionate attitude toward the self (“I still like who I am”). The FSCSRS starts with a first probe statement: “When things go wrong for me (…).” The participants respond on a 5-point scale (ranging from 0 = “not at all like me” to 4 = “extremely like me”) on a series of questions (e.g., “I think I deserve my self-criticism,” “There is a part of me that puts me down,” and “I find it easy to forgive myself”). The statements of the FSCSRS were derived from clinical work with depressed people where Pinto–Gouveia had noted some typical thoughts depressed patients offered about their self-criticism and ability to self-reassure (Castilho et al., 2015). Higher mean scores reflect a greater sense of inadequacy, hated-self, and self-reassurance (scores 0–5). The internal reliability of “Inadequate-self” was α = 0.92, of “Hated-self” was α = 0.74, and of “Reassuring-self” was α = 0.92.

### Depression Anxiety Stress Scale

Depression, anxiety, and stress levels of participants were measured using the Portuguese version of depression anxiety stress scale (DASS-21) (Lovibond and Lovibond, 1995; Pais-Ribeiro et al., 2004). The DASS-21 is a self-report scale with 21 items, seven for each subscale (e.g., “I felt that life was meaningless,” “I felt I was close to panic,” and “I found it difficult to relax”). The “Depression scale” measures symptoms of dysphoria, hopelessness, devaluation of life, self-deprecation, lack of interest/involvement, anhedonia, and inertia. The “Anxiety scale” measures autonomic arousal, skeletal muscle effects, situational anxiety, and subjective experience of anxious affect. Finally, the “Stress scale” assesses difficulty relaxing, nervous arousal, and being easily upset/agitated, irritable/over-reactive, and impatient. The participants rate the extent to which they have experienced each symptom over the past week, on a 4-point scale (0 = “did not apply to me at all” to 3 = “applied to me very much, or most of the time”). The sum scores for DASS dimensions were computed and, for comparison with the original DASS, scores were multiplied by two. Higher scores indicate more frequent anxiety, depression, and stress symptoms (scores 0–42). It must be highlighted that we intentionally used a dimensional score of depressive and anxious symptoms to capture symptoms in a continuum of severity, rather than a clinical diagnosis of PPD or anxiety disorders associated with postpartum. The structure of the Portuguese version of DASS-21 was identical to the original version, with the same items on the same scale. Good internal reliability was obtained for all subscales [depression (α = 0.87), anxiety (α = 0.82), and stress (α = 0.90)].

### Postpartum Bonding Questionnaire

Mother–infant bonding was assessed by the Portuguese short version of the postpartum bonding questionnaire (PBQ) (Brockington et al., 2001; Nazaré et al., 2012). The PBQ is a self-report, 12-item scale that assesses the mother's feelings or attitudes toward her baby (e.g., “I feel distant from my baby” and “I love to cuddle my baby”). The participants were asked to rate how often they agreed with these statements reflecting their experience on a 6-point scale ranging from 0 (always) to 5 (never), with reverse coding of positive statements. Higher mean scores indicate greater problems of mother–infant bonding. Through confirmatory factorial analysis the authors in the Portuguese version of the scale analyzed six models, which were based on previous PBQ studies (Nazaré et al., 2012). A 12-item structure that corresponded to the first factor of the original structure of the scale, named impaired mother–infant bonding (Brockington et al., 2001), was identified as having the best fit to their data, with good levels of internal as well as temporal consistency, along with adequate values of convergent and discriminant validity. In this study, a good internal reliability was obtained for the postpartum bonding scale (α = 0.75).

### Procedure

This study is part of a larger research project dedicated to risk and protective factors for (un)adjustment to motherhood. The study was previously approved by the Ethics and Deontology Committee of the School of Psychology and Life Sciences from University Lusofona. The data collection occurred between February and March of 2020. A non-probabilistic sampling was delivered based on a snow-ball method. The study comprised an online survey made in Typeform and was advertised in internet forums of mothers and on Facebook groups dedicated to maternal topics.

### Statistical Analysis

Data analyses were performed using IBM SPSS (v. 28). Descriptive analyses were conducted for sociodemographic and study variables. Zero-order correlations between the study variables were computed. Effect sizes of correlations were based on Cohen's guidelines (1988; small: Pearson's *r* = 0.10; medium: *r* = 0.30; and strong: *r* = 0.50).

**Figure 1:**
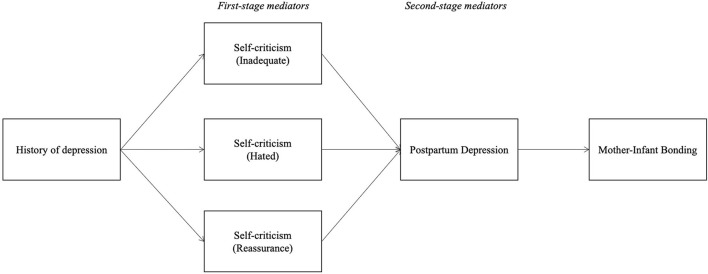
Conceptual mediation model for the presumed influence of history of depression symptoms on mother–infant bonding, through self-criticism and PPD symptoms.

**Figure 2:**
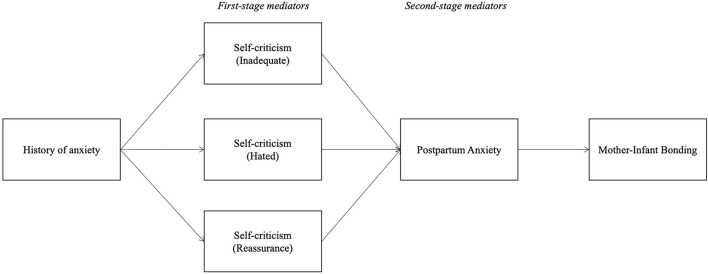
Conceptual mediation model for the presumed influence of history of anxiety symptoms on mother–infant bonding, through self-criticism and postpartum anxiety symptoms.

To test our hypotheses and examine whether the main effects of history of depression symptoms and anxiety symptoms on mother–infant bonding are mediated by self-criticism (inadequate, hated, and reassuring self), as well as postpartum negative affect (depression and anxiety symptoms), we tested two parallel and serial mediation models using PROCESS version 4.0 for IBM SPSS Statistics (Model 80; Hayes, 2018). In the models performed, history of depression and anxiety symptoms were entered as independent variables (each tested independently), self-criticism dimensions were the parallel first step mediators, postpartum negative affect (depression or anxiety) was the serial second step mediator and bonding the dependent variable. Accordingly, the first tested model evaluated the indirect effect of the history of depression symptoms (before and during pregnancy) on mother–infant bonding through the three dimensions of self-criticism (as first step mediators), and PPD symptoms (as a second step mediator; see Figure 1). The second tested model checked the indirect effect of the history of anxiety symptoms (before and during pregnancy) on mother–infant bonding through the three dimensions of self-criticism (as first step mediators) and postpartum anxiety symptoms (as second step mediator; see Figure 2). Given that comorbidity between depression and anxiety is common (Kalin, 2020), to control for these overlapping symptoms and consider the variability caused by the history of depression and anxiety symptoms before and during pregnancy, we included these variables as covariates in the analysis. As such, in Model 1, we controlled for the effect of history of anxiety symptoms and in Model 2, we controlled for the effect of history of depression symptoms. Additionally, we controlled for the effect of infants' age, mothers' age, income, and gestational complications. Indirect effects were tested through a bootstrapping procedure, including 5,000 bootstrap and 95% bias-corrected, and accelerated confidence intervals. Indirect effects were considered significant when zero was not included in the bootstrap 95% Confidence Interval.

## Results

Correlations between study variables are shown in Table 2. All study variables were significantly correlated with each other. Results show a high association between having a history of depression and anxiety symptoms, before and during pregnancy. History of depression and anxiety symptoms were positively correlated with the dimensions of self-criticism inadequate-self and hated-self, but negatively with reassuring self. High inadequate-self and hated-self and lower reassuring self-criticism was associated with higher levels of PPD and anxiety symptoms. Moreover, higher levels of self-criticism (hated and inadequate) were associated with higher problems in bonding. In the opposite direction, reassuring-self was negatively associated with bonding problems. Depression symptoms before and after partum, and anxiety symptoms before and after partum, were positively related to problems in mother–infant bonding.

**Table 2:** Correlations and descriptive statistics of history of depression and anxiety symptoms, self-criticism, postpartum negative affect, and mother–infant bonding.

**Study variables**	**1**.	**2**.	**3**.	**4**.	**5**.	**6**.	**7**.	**8**.	**9**.	**10**.	***M (SD)***
1. History of depression symptoms	–										2.44 (1.05)
2. History of anxiety symptoms	0.75***	–									2.27 (1.03)
3. Self-criticism (inadequate-self)	0.37***	0.39***	–								1.24 (0.89)
4. Self-criticism (hated-self)	0.30***	0.32***	0.60***	–							0.29 (0.60)
5. Self-criticism (reassuring self)	−0.40***	−0.40***	−0.49***	−0.41***	–						1.89 (0.92)
6. PPD symptoms	0.38***	0.38***	0.60***	0.61***	−0.44***	–					4.82 (6.54)
7. Postpartum anxiety symptoms	0.36***	0.42***	0.40***	0.44***	−0.36***	0.67***	–				3.82 (5.55)
8. Mother–infant bonding	0.22***	0.23***	0.30***	0.22***	−0.18**	0.34***	0.25***	–			0.37 (0.34)
9. Infants' age (months)	−0.02	0.01	−0.01	−0.03	0.02	0.05	0.09*	0.12**	–		8.57 (6.51)
10. Mothers' age (years)	−0.09*	−0.09*	−0.05	−0.10*	0.08	−0.09*	−0.09*	−0.07	0.11*	–	32.71 (5.19)

*p <0.05; **p <0.01; ***p <0.001.

### History of Depression and Mother–Infant Bonding

In mediational analyses, findings (Table 3) show that having a history of depression symptoms influences the dimensions of self-criticism as it increases mothers' sense of inadequate-self and hated-self, while it reduces the levels of reassuring-self. Both harsh self-criticism dimensions, inadequate-self and hated-self, are associated with increased PPD symptoms. Reassuring-self is associated with decreased PPD symptoms. Finally, when controlling for all the variables in the model, PPD symptoms are positively associated with impaired mother–infant bonding, and the direct effect of history of depression symptoms on mother-infant bonding decreases and becomes non-significant.

**Table 3:** Standardized regression coefficients (β), unstandardized regression coefficients (b), standard errors (SE), and model summary information for the tested serial–parallel mediation Model 1.

	**Inadequate-self**				**Hated-self**				**Reassuring-self**				**PPD symptoms**				**Mother–infant bonding**				**Mother–infant bonding**			
																	**(Total effect model)**				**(Serial–parallel mediation model)**			
**Antecedent**	**β**	***b***	***SE***	***P***	**β**	***b***	***SE***	***P***	**β**	***b***	***SE***	***p***	**β**	***b***	***SE***	***p***	**β**	***b***	***SE***	***p***	**β**	***b***	***SE***	***p***
History of depression symptoms (IV)	0.19	0.17	0.05	0.001	0.134	0.08	0.04	0.029	−0.23	−0.20	0.05	0.000	0.09	0.29	0.15	0.058	0.15	0.05	0.20	0.018	0.08	0.03	0.02	0.204
Inadequate-self (M1)	–	–	–	–	–	–	–	–	–	–	–	–	0.26	0.94	0.16	0.000	–	–	–	–	0.12	0.05	0.02	0.032
Hated-self (M2)	–	–	–	–	–	–	–	–	–	–	–	–	0.37	1.99	0.22	0.000	–	–	–	–	−0.02	−0.01	0.03	0.770
Reassuring-self (M3)	–	–	–	–	–	–	–	–	–	–	–	–	−0.10	−0.36	0.13	0.008	–	–	–	–	0.02	0.01	0.02	0.706
PPD symptoms (M4)	–	–	–	–	–	–	–	–	–	–	–	–	–	–	–	–	–	–	–	–	0.25	0.03	0.01	0.000
Constant	–	0.40	0.26	0.120	–	−0.20	0.18	0.261	–	2.50	0.26	0.000	–	0.77	0.83	0.354	–	−0.13	0.10	0.216	–	−0.17	0.11	0.107
History of anxiety symptoms (cov)	0.25	0.22	0.05	0.000	0.20	0.12	0.04	0.001	−0.22	−0.19	0.05	0.000	0.05	0.14	0.15	0.351	0.14	0.05	0.02	0.025	0.07	0.02	0.02	0.282
Mothers' age (cov)	−0.02	−0.004	0.01	0.617	−0.05	−0.01	0.00	0.214	0.02	0.00	0.01	0.624	−0.02	−0.01	0.02	0.552	0.06	0.04	0.00	0.153	0.07	0.00	0.00	0.073
Infants' age (cov)	−0.01	−0.001	0.01	0.802	−0.02	0.00	0.00	0.618	0.01	0.00	0.01	0.825	0.07	0.04	0.02	0.020	0.11	0.01	0.00	0.010	0.09	0.00	0.00	0.021
Gestational complications (cov)	0.01	0.02	0.07	0.837	0.00	0.00	0.05	0.931	−0.01	−0.01	0.07	0.865	0.01	0.09	0.21	0.665	−0.03	−0.02	0.03	0.446	−0.04	−0.02	0.03	0.362
Income (cov)	0.03	0.02	0.02	0.478	−0.09	−0.03	0.02	0.040	0.08	0.04	0.02	0.042	−0.05	−0.10	0.06	0.135	0.12	0.02	0.01	0.005	0.14	0.03	0.01	0.001
	*R*^2^= 0.17				*R*^2^ = 0.12				*R*^2^ = 0.19				*R*^2^ = 0.48				*R*^2^ = 0.10				*R*^2^ = 0.17			
	*F*_(6, 535)_ = 18.59, *p* < 0.001				*F*_(6, 535)_ = 12.23, *p* < 0.001				*F*_(6, 535)_ = 21.14, *p* < 0.001				*F*_(9, 532)_ = 54.88, *p* < 0.001				*F*_(6, 535)_ = 9.44, *p* < 0.001				*F* (10,531) = 11.19, *p* < 0.001			

**Figure 3:**
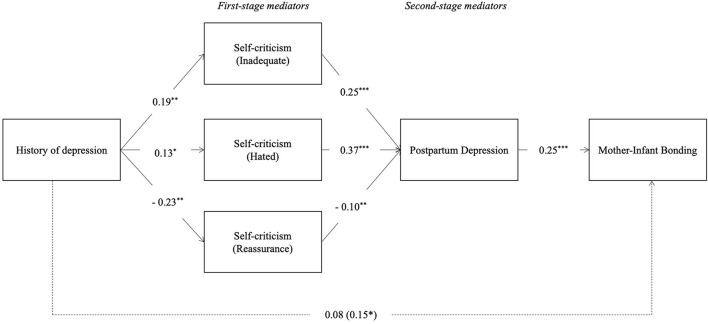
Serial–parallel mediation model with standardized path coefficients: History of depression symptoms, inadequate-self, hated-self and reassuring-self dimensions of self-criticism, PPD Symptoms and mother–infant bonding (M1). *p < 0.05, **p < 0.01, ***p < 0.001. The coefficients of the total effects appear in parentheses. Dashed lines are not-significant paths.

The model testing the indirect effect of the history of depression symptoms on mother–infant bonding (Figure 3) showed significant indirect effects through the chain of mediators considered (see Table 4). As such, we found the following indirect effects of the serial mediation models. (1) First, through the self-criticism dimension of inadequate-self and through PPD symptoms; (2) second, through the self-criticism dimension of hated-self, and through PPD symptoms; and (3) finally through the self-criticism dimension of reassuring-self followed by PPD symptoms.

**Table 4:** Standardized coefficients (β), unstandardized coefficients (b), unstandardized boot standard errors, and boot 95% confidence intervals of the unstandardized indirect effects of history of depression symptoms on mother–infant bonding, through inadequate-self, hated-self, reassuring-self, PPD symptoms (Model 1).

**Specific indirect effect (mediators)**	**β**	***b***	**Boot SE**	**Boot 95% CI**
History of depression symptoms → Inadequate-self → Mother–infant bonding	0.023	0.008	0.006	[−0.001, 0.020]
History of depression symptoms → Hated-self → Mother–infant bonding	−0.002	−0.001	0.003	[−0.007, 0.008]
History of depression symptoms → Reassuring-self → Mother–infant bonding	−0.004	−0.001	0.003	[−0.008, 0.005]
History of depression symptoms → PPD symptoms → Mother–infant bonding	0.023	0.007	0.005	[−0.0001, 0.018]
History of depression symptoms → Inadequate-self → PPD symptoms → Mother–infant bonding	0.012	0.004	0.002	[0.001, 0.009]
History of depression symptoms → Hated-self → PPD symptoms → Mother–infant bonding	0.012	0.004	0.003	[0.0002, 0.010]
History of depression symptoms → Reassuring-self → PPD symptoms → Mother–infant bonding	0.006	0.002	0.001	[0.0003, 0.005]

### History of Anxiety and Mother–Infant Bonding

**Figure 4:**
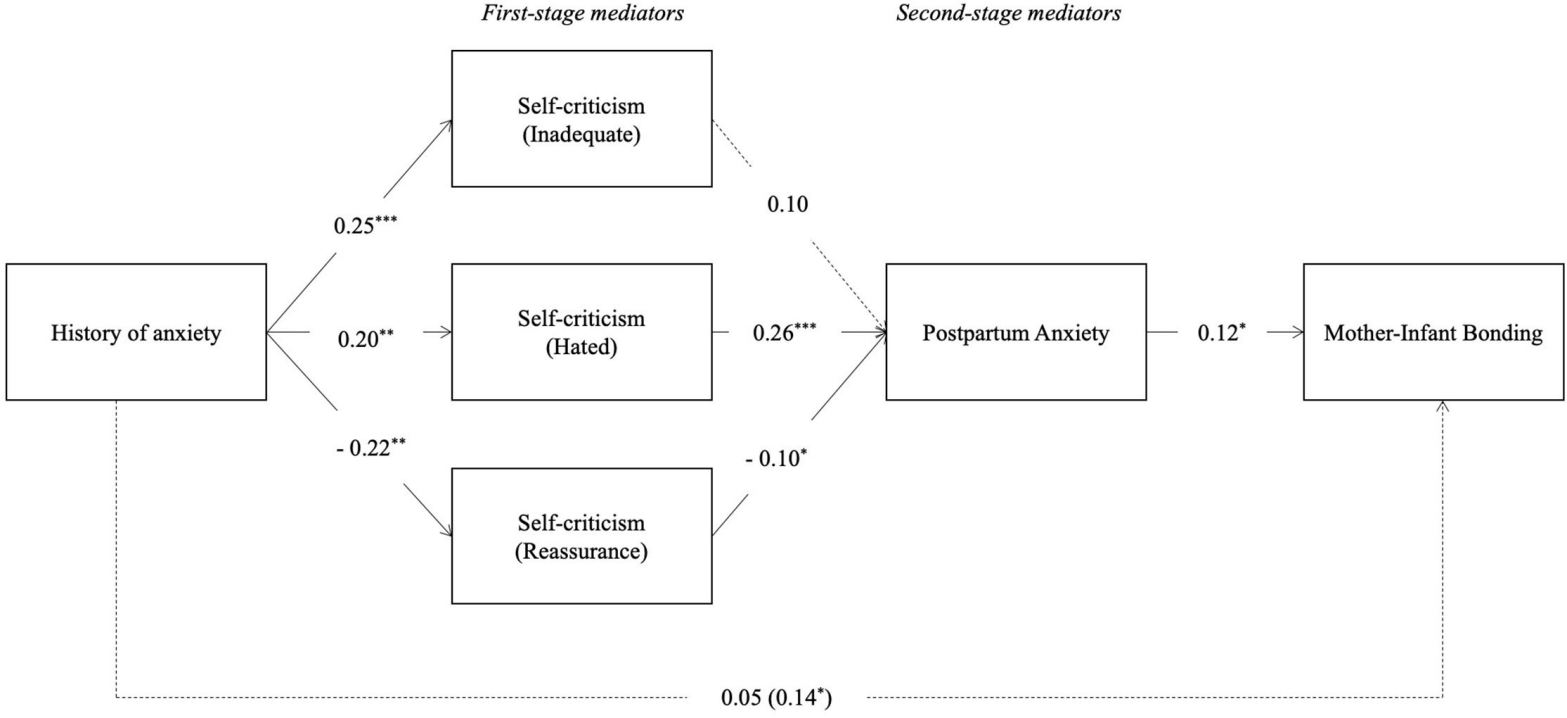
Serial–parallel mediation model with standardized path coefficients: History of anxiety symptoms, inadequate-self, hated-self, and reassuring-self dimensions of self-criticism, postpartum anxiety symptoms and mother–infant bonding (M1). *p < 0.05, **p < 0.01, ***p < 0.001. The coefficients of the total effects appear in parentheses. Dashed lines are not-significant paths.

Results (Table 5) show a significant effect of mother's history of anxiety symptoms on the three dimensions of self-criticism, as it increases mother's sense of inadequate-self and hated-self, while it reduces the levels of reassuring self-criticism. Different from Model 1, in Model 2, only the self-criticism dimensions of the hated-self and the reassuring-self are significantly associated with postpartum anxiety symptoms, although in different directions. Accordingly, the greater the feelings of hated self, the greater the postpartum anxiety, while greater levels of mother's reassurance are associated with lower levels of postpartum anxiety symptoms. Finally, regarding the second-step mediator, postpartum anxiety is positively associated with impaired mother–infant bonding. When controlling for all the mediators in the model, in relation to the total effect, the direct effect of the history of anxiety symptoms on mother–infant bonding decreases and becomes non-significant. Also, when testing the indirect effects of the history of anxiety symptoms on mother–infant bonding (Figure 4) through the overall chain of mediators, there are no significant indirect effects (see Table 6). Results show that inadequate-self is, alone, a significant mediator of the relation between history of anxiety symptoms and infant–mother bonding.

**Table 5:** Standardized regression coefficients (β), unstandardized regression coefficients (*b*), standard errors (SE), and model summary information for the tested serial–parallel mediation Model 2.

	**Inadequate-self**				**Hated-self**				**Reassuring-self**				**Postpartum Anxiety**				**Mother–infant bonding**				**Mother–infant bonding**			
																	**(Total effect model)**				**(Serial–parallel mediation model)**			
**Antecedent**	**β**	***b***	***SE***	***p***	**β**	***b***	***SE***	***p***	**β**	***b***	***SE***	***p***	**β**	***b***	***SE***	***p***	**β**	***b***	***SE***	***p***	**β**	***b***	***SE***	***p***
History of anxiety symptoms (IV)	0.25	0.22	0.05	0.00	0.20	0.12	0.04	0.001	−0.22	−0.19	0.05	0.000	0.221	0.60	0.15	0.00	0.14	0.05	0.02	0.025	0.050	0.02	0.02	0.43
Inadequate-self (M1)	–	–	–	–	–	–	–	–	–	–	–	–	0.096	0.30	0.15	0.05	–	–	–	–	0.172	0.06	0.02	0.00
Hated-self (M2)	–	–	–	–	–	–	–	–	–	–	–	–	0.262	1.21	0.21	0.00	–	–	–	–	0.043	0.02	0.03	0.41
Reassuring-self (M3)	–	–	–	–	–	–	–	–	–	–	–	–	−0.096	−0.29	0.13	0.28	–	–	–	–	0.005	0.00	0.02	0.92
Postpartum anxiety symptoms (M4)	–	–	–	–	–	–	–	–	–	–	–	–	–	–	–	–	–	–	–	–	0.121	0.01	0.01	0.01
Constant	–	0.40	0.26	0.12	–	0.20	0.18	0.261	–	2.50	0.26	0.000	–	0.49	0.80	0.54	–	0.13	0.10	0.216	–	−0.16	0.11	0.14
History of depression symptoms (cov)	0.19	0.17	0.05	0.00	0.13	0.08	0.04	0.029	−0.23	−0.20	0.05	0.000	0.033	0.09	0.15	0.54	0.15	0.05	0.02	0.018	0.096	0.03	0.02	0.11
Mothers' age (cov)	−0.02	0.00	0.01	0.62	−0.05	−0.01	0.00	0.214	0.02	0.00	0.01	0.624	−0.058	−0.03	0.02	0.11	0.06	0.00	0.00	0.153	0.076	0.01	0.00	0.07
Infants' age (cov)	−0.01	0.00	0.01	0.80	−0.02	0.00	0.00	0.618	0.01	0.00	0.01	0.825	0.115	0.05	0.02	0.00	0.11	0.01	0.00	0.010	0.097	0.01	0.00	0.02
Gestational complications (cov)	0.01	0.01	0.07	0.84	0.00	0.00	0.05	0.931	−0.01	−0.01	0.07	0.865	0.104	0.59	0.20	0.00	−0.02	−0.02	0.03	0.446	−0.046	−0.03	0.03	0.26
Income (cov)	0.03	0.02	0.02	0.48	−0.09	−0.03	0.02	0.040	0.08	0.04	0.02	0.042	−0.011	−0.02	0.06	0.78	0.12	0.02	0.01	0.005	0.125	0.02	0.01	0.00
	*R*^2^= 0.17				*R*^2^ = 0.12				*R*^2^ = 0.19				*R*^2^ = 0.33				*R*^2^ = 0.10				*R*^2^ = 0.15			
	*F*_(6, 535)_ = 18.59, *p* < 0.001				*F*_(6, 535)_ = 12.23, *p* < 0.001				*F*_(6, 535)_ = 21.14, *p* < 0.001				*F*_(9, 532)_ = 28.56, *p* < 0.001				*F*_(6, 535)_ = 9.44, *p* < 0.001				*F* (10,531) = 9.53, *p* < 0.001			

**Table 6:** Standardized coefficients (β), unstandardized coefficients (*b*), unstandardized boot standard errors, and boot 95% confidence intervals of the unstandardized indirect effects history of anxiety symptoms on mother–infant bonding, through inadequate-self, hated-self, reassuring-self, postpartum anxiety symptoms (Model 2).

**Specific indirect effect (mediators)**	**β**	***b***	**Boot SE**	**Boot 95% CI**
History of anxiety symptoms → Inadequate-self → Mother–infant bonding	0.041	0.014	0.007	[0.003, 0.030]
History of anxiety symptoms → Hated-self → Mother–infant bonding	0.009	0.003	0.005	[−0.007, 0.012]
History of anxiety symptoms → Reassuring-self → Mother–infant bonding	−0.001	−0.001	0.003	[−0.007, 0.007]
History of anxiety symptoms → Postpartum anxiety symptoms → Mother–infant bonding	0.027	0.009	0.006	[−0.0002, 0.023]
History of anxiety symptoms → Inadequate-self → Postpartum anxiety symptoms → Mother–infant bonding	0.003	0.001	0.001	[−0.0004, 0.003]
History of anxiety symptoms → Hated-self → Postpartum anxiety symptoms → Mother–infant bonding	0.006	0.002	0.002	[−0.0001, 0.006]
History of anxiety symptoms → Reassuring-self → Postpartum anxiety symptoms → Mother–infant bonding	0.003	0.001	0.001	[−0.0000, 0.003]

## Discussion

This study analyzed if maternal history of depression symptoms predicted mother–infant bonding and if this relation was mediated sequentially by mother's self-criticism and present symptoms of depression. The same model was explored for anxiety, including history of anxiety symptoms as a predictor and present symptoms as mediator.

The results evidenced a high association between having a history of depression and anxiety symptoms, before and during pregnancy, which is in line with the vast literature that describes the overlapping and comorbid relations between these two frequent psychological conditions. Similarly, the positive associations were found between the history of depression symptoms and the PPD symptoms, and between the history of anxiety symptoms and postpartum anxiety symptoms, highlighting the continuity of these risk factors during the peripartum period. These findings were in line with previous studies that showed the co-occurrence and the positive association between anxiety and depressive symptoms during PPD (e.g., Heron et al., 2004).

As for the first model of our study, the results have demonstrated that the history of depression symptoms showed significant indirect effects on mother–infant bonding through the chain of mediators, in line with the studies showing that mothers with a psychiatric history are at a higher risk of PPD symptoms and bonding problems (Lefkowitz et al., 2010; de Kruijff et al., 2019; Nakić Radoš et al., 2020; Tolja et al., 2020).

In addition, findings show that having a history of depression symptoms is associated with higher levels of inadequate-self and hated-self, and lower levels of self-reassurance, which is consistent with previous studies showing positive associations of inadequate-self and hated-self with psychopathology (Castilho et al., 2017) and a negative association of self-reassurance with psychopathology (Gilbert et al., 2004; Werner et al., 2019). The relationship between the inadequate self, hated-self and reassuring self, and PPD symptoms showed a similar pattern. This is consistent with the existing research that showed that the self-criticism had a negative effect on PPD symptoms (e.g., Vliegen and Luyten, 2009) and on those feelings of self-inadequacy mediated the stress-depression relationship (e.g., Kotera et al., 2021). Also, a stronger positive relationship between hated-self and depression was found, which is consistent with evidence showing hated-self to be consistently more highly associated with psychopathology than the inadequate self (Gilbert et al., 2004; Castilho et al., 2017; Werner et al., 2019).

Finally, higher levels of postpartum depressive symptoms were associated with higher problems in bonding, which confirms previous evidence from other studies (Edhborg et al., 2011; Dubber et al., 2015; Nakić Radoš et al., 2020; Tolja et al., 2020; Handelzalts et al., 2021). This finding is important since it facilitates understanding of how maternal depression might impact bonding and further outcomes on infant health. Maternal depression might affect maternal bonding (Noorlander et al., 2008) and might lead to an insensitive caretaking environment (Nicol-Harper et al., 2007; Kaitz et al., 2010; Müller et al., 2016). Insensitive caretaking which can be seen in PPD symptoms might be affecting the difficulties in self-regulation of the infant (Manian and Bornstein, 2009).

As for the second model of our study, history of anxiety symptoms showed no significant indirect effects on mother–infant bonding through the overall chain of mediators. Furthermore, in our study, the direct effect of history of anxiety symptoms decreases and becomes non-significant on mother–infant bonding. Considering that there are very few studies examining the relationship between postpartum anxiety symptoms and bonding, and studies examining the relationship between the history of anxiety symptoms and bonding are even rarer and have heterogeneous results (Dubber et al., 2015; Göbel et al., 2018), this finding can be considered as a reflection of another aspect of maternal feelings on bonding before and during pregnancy. It can be evaluated that anxiety before and during pregnancy might be somehow functional in terms of bonding during the transition to parenthood (e.g., serving to protect the baby) (Figueiredo and Conde, 2011). Therefore, this finding between the history of anxiety and bonding may have been obtained. With respect to the link between postpartum anxiety symptoms and bonding, as expected, higher levels of postpartum anxiety symptoms were associated with higher problems in bonding, which confirms previous evidence from other studies (Edhborg et al., 2011; Tietz et al., 2014; Dubber et al., 2015). Mothers experiencing anxiety might show more difficulty in self-regulating, and in interacting sensitively and regulating the child (Feldman et al., 1997; Tietz et al., 2014).

In addition, consistent with previous studies on the links between self-criticism and psychopathology (Gilbert et al., 2004; Castilho et al., 2017; Werner et al., 2019), having a history of anxiety symptoms was associated with higher levels of inadequate-self and hated-self, and lower levels of self-reassurance. The same was true with regards to the relationship between the inadequate self and reassuring self, and postpartum anxiety symptoms, which is in line with previous studies showing a negative effect of self-criticism in anxiety in the postpartum period (e.g., Vliegen and Luyten, 2009; Kotera et al., 2021). Therefore, individual attempts to cope with one's feelings of inadequacy can play an important role in the experience of anxiety in the postpartum period. Furthermore, no relationship regarding hated-self and anxiety was found. This is somewhat surprising, given evidence showing that the hated-self is more detrimental to mental health than the inadequate self (Gilbert et al., 2004; Castilho et al., 2017; Werner et al., 2019). Studies investigating the association between self-criticism and psychopathology have mostly used clinical samples (McIntyre et al., 2018; Werner et al., 2019); therefore, the fact that we used a community sample might contribute to explaining these unexpected findings regarding anxiety. Further studies that are conducted with non-clinical samples that explore the relationship between self-criticism and anxiety postpartum are needed.

### Limitations and Recommendations for Future Studies

The studies presented several limitations that must be addressed. First, since this was a cross-sectional study, no causality could be inferred based on the analyses performed. Also, although the participants were instructed the exact time point to answer, the collection of data in one point could be considered as a limitation in terms of observer bias, perhaps calling into question if perhaps maternal perceptions of their previous mood as well as cognitions about the self and bonding with their child are not a function of their mood at the time of data collection. Therefore, some caution is needed when interpreting our findings. Future studies should include longitudinal designs to overcome this limitation. For instance, self-criticism, postpartum negative symptoms, and perception of infant–mother bonding of mothers with negative symptoms before and during pregnancy should be assessed 3–9 months after childbirth and, ideally, 1–2 years after childbirth to infer the possible causality and the identification of trajectories related to the impact of the history of depression and anxiety symptoms across diverse phases of postpartum period. Second, our non-probabilistic sampling procedures (i.e., convenience and snowball techniques and data collection based on social application's advertisements) might have influenced the characteristics of the sample and attracted mothers more motivated to respond to this large protocol, more digitally proficient, and with less particular impairments (psychical or neuropsychological). Also, the discrepancy between Cronbach α between the hated-self (0.74) and the inadequate self (0.92) should be noted, even though it is coherent with previous studies (e.g., Castilho et al., 2015).

Third, although the literature has highlighted the existence of high comorbidity between PPD and anxiety symptoms, our results are only focused on symptoms of PPD and anxiety, separately. Future studies could consider testing similar models with symptoms of anxiety and depression together. Moreover, self-report measures might be biased by social desirability, especially concerning mother–infant bonding.

Fourth, certain key variables are highly correlated as given in the following: History of depression symptoms and history of anxiety symptoms; PPD and postpartum anxiety symptoms; PPD symptoms and hated-self; PPD symptoms and inadequate-self. Also, although the participants were instructed the exact time point to answer, the collection of data in one point can be considered as a limitation in terms of observer bias, perhaps calling into question if perhaps maternal perceptions of their previous mood as well as cognitions about the self and bonding with their child are not a function of their mood at the time of data collection. Therefore, some cautions are needed when interpreting our findings.

Fifth, the factors concerning the context (e.g., partner's, family's or professional's support, and infant temperament and characteristics) were left out of the analyses. Future research must include their possible impact on maternal negative affect and on mother–infant bonding during the postpartum period. In addition, only intrapersonal variables are considered in the models, neither contextual nor “child” variables are included. Future research should take this into account and have more information about and from other informants and sources.

Also, the future research should test whether the proposed models apply to both common and clinically significant levels of anxiety and depression. Furthermore, the symptoms across the postpartum period, limited data exist about the stability and specific trajectories of these symptoms, and even less about the evolution of anxiety symptoms during postpartum. For that reason, the wide range of infants' ages requires caution in the interpretation of the results. Future research should consider a limited range of age, but also should characterize the pathways associated with emotional symptomatology across the postpartum period.

Finally, our study focused on depressive and anxious symptoms and on different types of self-criticism as mediating mechanisms. As such, our results should explore the cognitions, the coping mechanisms, and the emotions, associated with self-criticism, that are more prevalent in women with both depression and anxiety symptoms, and the differences among them. Moreover, the role of self-reassuring styles and self-compassion should be studied as possible protective factors for anxiety and depression in postpartum, and for the quality of mother–child bonding. Further exploration on the mechanisms through which self-critical thinking might impact psychopathology in postpartum would be important, especially regarding anxiety in which knowledge is still limited.

### Strengths and Implications

This study adds to the existing research by examining both retrospectively self-reported levels of depression and anxiety symptoms before and during pregnancy, and the current (postpartum) levels of depression and anxiety symptoms in a large community sample in Portugal. The previous studies of self-criticism have been implicated in a range of psychopathologies (McIntyre et al., 2018). Similarly, our findings also evidence the possible transdiagnostic role of self-criticism in the comprehension and maintenance of anxiety and depression during postpartum and bonding. Furthermore, we controlled the effect of depression and anxiety on each model, evidencing the differential contribution of anxiety and depression to bonding problems. Thus, they should be addressed as comorbid, despite being distinct phenotypic conditions.

The results from this study have important specific clinical implications. Given that the history of both depression and anxiety symptoms have predicted negative affect and bonding, concrete screening assessment delivered on mental care and general health institutions during pregnancy should address the existence of depressive and anxious symptoms prior and during pregnancy to help women at risk of postpartum distress and provide specific interventions. Furthermore, our findings suggest that decreasing maternal self-criticism should be targeted in preventive and therapeutic psychological interventions, and self-reassurance, which represents a self-compassionate attitude, should be promoted as a buffer mechanism to reduce the incidence of negative symptoms and bonding problems during the postpartum period. In this way, cultivating a self-accepting, mindful and non-judgmental mindset might help future and recent new mothers to adapt to changes and difficulties from this period with lower levels of self-criticism and less negative affect. Feeling less depressed and anxious might prevent bonding difficulties and less risk factors for mental health and wellbeing in mothers and children.

## Conclusions

Maternal depression and other psychological problems have been described in literature as having considerable consequences on bonding during the postpartum period and afterward. This study added new insights on this previous evidence, revealing that the quality of mother–infant bonding in postpartum might be affected by the history of depression symptoms in mothers, but especially, that self-criticism and consequently the depressive symptoms might play a role in this relation. Further, the history of anxiety symptoms also has an impact on bonding but only is mediated by hated-self and, in an opposite way, by reassuring self. Our results highlight the importance of assessing previous history of maternal psychological symptoms and psychopathology, as they might represent an important risk factor for bonding in the postpartum period. Further, interventions might need to promote more self-compassionate attitudes in mothers to prevent maladaptation after the birth of a child.

## Data Availability Statement

The raw data supporting the conclusions of this article will be made available by the authors, without undue reservation.

## Ethics Statement

The studies involving human participants were reviewed and approved by Ethical and Deontological Committee for Scientific Research of the School of Psychology and Life Sciences (CEDIC). The patients/participants provided their written informed consent to participate in this study.

## Author Contributions

AB and LC contributed to the conception and design of the study. AB, SA, and LC organized the database. LC and ÁS performed the statistical analysis and wrote sections of the manuscript. AB, SA, and BK wrote the first draft of the manuscript. All authors contributed to manuscript revision, read, and approved the submitted version.

## Funding

The authors received funding support from FCT (HEI-Lab, UIDB/05380/2020), Lusófona University for the open access publication fee.

## Conflict of Interest

The authors declare that the research was conducted in the absence of any commercial or financial relationships that could be construed as a potential conflict of interest.

## Publisher's Note

All claims expressed in this article are solely those of the authors and do not necessarily represent those of their affiliated organizations, or those of the publisher, the editors and the reviewers. Any product that may be evaluated in this article, or claim that may be made by its manufacturer, is not guaranteed or endorsed by the publisher.
